# Incidence of Self-Reported Diabetes in New York City, 2002, 2004, and 2008

**DOI:** 10.5888/pcd9.110320

**Published:** 2012-06-14

**Authors:** Bahman P. Tabaei, Shadi Chamany, Cynthia R. Driver, Bonnie Kerker, Lynn Silver

**Affiliations:** Author Affiliations: Shadi Chamany, Cynthia R. Driver, Bonnie Kerker, Lynn Silver, New York City Department of Health and Mental Hygiene, New York, New York.

## Abstract

**Introduction:**

Prevalence and incidence of diabetes among adults are increasing in the United States. The purpose of this study was to estimate the incidence of self-reported diabetes in New York City, examine factors associated with diabetes incidence, and estimate changes in the incidence over time.

**Methods:**

We used data from the New York City Community Health Survey in 2002, 2004, and 2008 to estimate the age-adjusted incidence of self-reported diabetes among 24,384 adults aged 18 years or older. Multiple logistic regression analysis was performed to examine factors associated with incident diabetes.

**Results:**

Survey results indicated that the age-adjusted incidence of diabetes per 1,000 population was 9.4 in 2002, 11.9 in 2004, and 8.6 in 2008. In multivariable-adjusted analysis, diabetes incidence was significantly associated with being aged 45 or older, being black or Hispanic, being overweight or obese, and having less than a high school diploma.

**Conclusion:**

Our results suggest that the incidence of diabetes in New York City may be stabilizing. Age, black race, Hispanic ethnicity, elevated body mass index, and low educational attainment are risk factors for diabetes. Large-scale implementation of prevention efforts addressing obesity and sedentary lifestyle and targeting racial/ethnic minority groups and those with low educational attainment are essential to control diabetes in New York City.

## Introduction

Recent epidemiologic studies indicate that the prevalence (1-3) and incidence (4-7) of diabetes among adults are increasing in the United States. From 1980 through 2009, the national prevalence of diabetes increased by as much as 144% (3), and the age-adjusted incidence increased by 151% (4). National survey data also show that diabetes incidence was higher with older age and increasing weight (body mass index [BMI] category), was higher among minority racial/ethnic groups, and was lower with higher education levels (6). The increases in prevalence and incidence may be a result of a true increase in diabetes incidence, use of an enhanced case definition and enhanced detection, an increase in the survival among prevalent diabetes cases, or some combination of these items (6). The extent to which any or all of these factors are at work is unknown because of limited population-based data on secular trends in diabetes incidence and survival.

Even less is known about the incidence of diabetes in smaller geographic areas such as counties and cities (8,9). A better understanding of local variations would provide a mechanism by which health care and public health professionals could assess the health of their communities and develop policies and allocate resources for the prevention and control of diabetes. However, routine epidemiologic surveillance of diabetes incidence at the local level is uncommon.

New York City (NYC), similar to the nation overall, has experienced an increase in diabetes prevalence over the past 2 decades. Between 1994 and 2004, the prevalence of age-adjusted self-reported diabetes among adults in NYC rose from 3.7% to 9.2% and has remained stable over the past several years. In 2010, the prevalence of diabetes in NYC was 9.3% (10,11) and the prevalence nationally was 8.7% (12). Because diabetes prevalence in NYC is higher than the national rate and the characteristics of prevalent cases do not always reflect recent incident cases, we sought to determine the estimated incidence of self-reported diabetes and associated risk factors in NYC between 2002 and 2008.

## Methods

### Sample

We used data from the 2002, 2004, and 2008 New York City Community Health Survey (CHS) to perform analyses. Data on diabetes incidence were not available from the 2003, 2005, 2006, 2007, 2009, and 2010 surveys. CHS methods are based on those used by the Behavioral Risk Factor Surveillance System (BRFSS) and have been previously described in detail (13). Briefly, by using a computer-assisted telephone interviewing system, CHS randomly samples approximately 10,000 noninstitutionalized adults aged 18 or older with residential (landline only) telephones annually (cell phones were included beginning in 2009). The CHS uses a cross-sectional stratified sample to obtain neighborhood-specific estimates of many health behaviors, health care access indicators, and health conditions to inform programs and policies. Neighborhood designations are determined by a zip code–based classification system developed and used by the United Hospital Fund (UHF) (14). Each record in the CHS was assigned a primary weight for the probability of selection (ie, number of adults in each household and number of residential telephone lines) and a poststratification weight to adjust the sample estimates to the composition of each UHF neighborhood, while taking into account the respondent’s age, sex, and race/ethnicity. A comparison of these weighted samples to the US Census 2000 population of NYC adults showed that the weighted CHS is representative of the adult population of NYC (13). No groups were oversampled in CHS. The overall cooperation rates (defined as the number of people who participated in the survey divided by the number of people in the sample who were contacted and identified as eligible [15]) were 69% in 2002, 63% in 2004, and 81% in 2008.

People were ineligible for our study if they were previously diagnosed with diabetes (n = 2,121), had a missing or don’t know/refused/not sure response for having diabetes (n = 70), or had a missing or don’t know/refused/not sure or invalid response for age or age at diagnosis (n = 238). Thus, the final sample size was 8,928 from 2002, 8,711 from 2004, and 6,745 from 2008. The final joint years sample size was 24,384.

### Measurements

All sampled adults in the 2002 and 2004 CHS (16,17) were asked “Have you ever been told by a doctor that you have diabetes?” All sampled adults in the 2008 CHS (18) were asked “Have you ever been told by a doctor, nurse or other health professional that you have diabetes?” Women who stated that they had been told they had diabetes only during pregnancy were not considered to be previously diagnosed with diabetes. Respondents reporting a diagnosis of diabetes in the 2002 and 2004 CHS were then asked “How old were you when you were told you have diabetes?” Respondents reporting a diagnosis of diabetes in the 2008 CHS were asked “How old were you when you were first told you have diabetes?” Duration of diagnosed diabetes was calculated by subtracting age at diagnosis from age at the time of interview. A value of 0 indicated that diabetes was diagnosed within the previous year and a value of 1 or more indicated that diabetes was previously diagnosed. However, using this method, some participants with a value of 1 would be misclassified if they had had a birthday between the date of diagnosis and date of interview. To adjust for this misclassification, half of those with a value of 1 were randomly selected and assumed to have had diabetes diagnosed within the previous year. The incidence of diabetes was then calculated using the number of the people who were diagnosed with diabetes within the previous year as the numerator and those who had not been diagnosed with diabetes as the denominator (6). Incidence was age adjusted according to the 2000 US standard population.

Self-reported race/ethnicity was measured by first asking “Are you Hispanic or Latino?” Respondents who stated they were not Hispanic or Latino were then asked, “Which one of these groups would you say best represents your race?” The options included white, black or African American, Asian, Native Hawaiian or other Pacific Islander, and American Indian or Alaska Native. Responses to the 2 questions were combined and recoded into the categories non-Hispanic white, non-Hispanic black, Hispanic, or other.

Self-reported height and weight were used to calculate body mass index (BMI, expressed as kg/m^2^). BMI was calculated as weight in kilograms divided by height in meters squared, and participants were classified into BMI categories (under/normal weight = BMI <25.0; overweight = BMI 25.0–29.9; obese = BMI ≥30.0 [13]).

Respondents’ education attainment was accessed by asking “What is the highest grade or year of school you completed?” Responses were categorized as more than high school graduate, high school graduate, and less than high school graduate.

Smoking status was measured by asking “Have you smoked at least 100 cigarettes in your entire life?” and if the response was yes, “Do you now smoke cigarettes every day, some days, or not at all?” Smoking status was categorized as never (people who answered no to the first question), current (people who answered yes to the first question and “Every day” or “Some days” to the second question), and former (people who answered yes to the first question and “Not at all” to the second question).

NYC neighborhood income level was obtained from Census 2000 data and defined as the percentage of the population in each neighborhood living below 200% of the federal poverty guidelines. The neighborhoods were categorized as high income (13%–<30%), medium income (30%–<43%), and low income (43%–70%).

### Statistical analysis

Crude and age-adjusted diabetes incidence was calculated for 2002, 2004, and 2008. The age-adjusted estimated incidence of self-reported diabetes was examined by sex, race/ethnicity, BMI category, education level, smoking status, and neighborhood income. Because of small sample size for individual CHS years, these estimates were examined for 2002, 2004, and 2008 combined to improve precision. Differences in proportions, percentages, and means were determined by the *t* test and χ^2^ test and were considered significantly different at *P* < .05. Relative standard errors (RSE), a measure of estimate precision, were calculated for all the incidence estimates (19). Estimates with an RSE higher than 30% or sample size (unweighted denominator for each analysis group) less than 50 are considered unreliable because of the variability in the data. There were no incidence estimates that fit these criteria.

To examine the factors associated with the estimated incidence of self-reported diabetes, logistic regression analysis was performed for the combined 2002, 2004, and 2008 surveys. Before building the multiple logistic regression model, the main effects were individually examined using simple logistic regression analysis to eliminate the nonsignificant predictors. Variables that were significantly associated with incident diabetes at *P* < .10 were considered for the multiple logistic regression model. A forward stepwise approach was used, and variables were entered in the order of their bivariate significance; those that were significant at the *P* < .05 level were left in the final model. Two-way interaction terms between significant variables were also tested; no interaction term was significant. Multicollinearity between the covariates was assessed using the Kendall’s τ-b correlation coefficient statistic for categorical data. There was no correlation higher than 0.24 between the covariates in the final multiple logistic regression model. The best model was assessed by using the likelihood ratio test. The fit of the model was assessed by the Hosmer-Lemeshow goodness of fit χ^2^ test. All statistical analyses were conducted using SAS software version 9.2 (SAS Institute, Inc, Cary, North Carolina) for data management and SAS-callable SUDAAN version 10.0 (Research Triangle Institute, Research Triangle Park, North Carolina) to obtain point estimates and confidence intervals.

## Results

In the CHS, the estimated crude incidence of self-reported diabetes per 1,000 population was 8.6 (95% confidence interval [CI], 6.7–11.0) for 2002, 10.9 (95% CI, 8.8–13.5) for 2004, and 7.5 (95% CI, 5.5–10.2) for 2008. The estimated age-adjusted incidence per 1,000 population was 9.4 (95% CI, 7.3–12.0) for 2002, 11.9 (95% CI, 9.6–14.6) for 2004, and 8.6 (95% CI, 6.3–11.6) for 2008. The differences in the estimated age-adjusted incidence across the years were not significant.

For the combined CHS, the age-specific self-reported incidence per 1,000 population was significantly higher among participants aged 45 years or older than among those younger than 45. The age-adjusted incidence per 1,000 population was significantly higher among non-Hispanic blacks and Hispanics than among whites, among overweight and obese people than among those at a normal weight, among high school graduates and below than among those with more than a high school diploma, and among people residing in low-income neighborhoods than among those residing in high-income neighborhoods (Table 1). Incident diabetes did not vary by sex or smoking status.

**Table 1:** Estimated Age-Adjusted Incidence of Self-Reported Diabetes, New York City Community Health Survey, Combined 2002, 2004, and 2008

Characteristic	Diabetes Incidence per 1,000 Population (95% CI)	*P* Value
**Total**	10.0 (8.6–11.5)	NA
**Age, y^a^**		
18–44	3.8 (2.8–5.1)	1 [Reference]
45–64	17.5 (14.2–21.6)	< .001
≥65	15.8 (12.3–20.4)	< .001
**Sex**		
Male	11.0 (8.8–13.8)	1 [Reference]
Female	9.0 (7.5–10.8)	.19
**Race/ethnicity**		
Non-Hispanic white	6.1 (4.7–7.9)	1 [Reference]
Non-Hispanic black	12.1 (9.4–15.6)	< .001
Hispanic	15.0 (11.4–19.6)	< .001
Other	11.2 (7.3–17.1)	.04
**BMI category^b^**		
Normal	6.2 (4.6–8.3)	1 [Reference]
Overweight	9.6 (7.4–12.5)	.03
Obese	18.6 (14.9–23.3)	< .001
**Education**		
>High school graduate	7.1 (5.6–9.0)	1 [Reference]
High school graduate	11.6 (9.0–15.0)	.009
<High school graduate	17.4 (13.2–22.8)	< .001
**Smoker**		
Never	9.4 (7.8–11.4)	1 [Reference]
Current	11.1 (8.0–15.5)	.42
Former	10.3 (7.6–14.0)	.64
**Neighborhood income level^c^**		
High	7.0 (5.2–9.4)	1 [Reference]
Medium	9.3 (7.2–11.9)	.15
Low	14.4 (11.6–17.9)	< .001

Abbreviation: CI, confidence interval, NA, not applicable; BMI, body mass index.

^a^ Age-specific groups are not age-adjusted.

^b^ BMI was calculated as weight in kilograms divided by height in meters squared, and participants were classified into BMI categories (under/normal weight = BMI <25.0; overweight = BMI 25.0–29.9; obese = BMI ≥30.0) (13).

^c^ Neighborhood income level was obtained from Census 2000 data and defined as the percentage of the population in each neighborhood living below 200% of the federal poverty guidelines. The neighborhoods were categorized as high income (13%–<30%), medium income (30%–<43%), and low income (43%–70%).

Multivariable analysis included only those variables with a *P* < .10 in the bivariate analysis: age (*P* < .001), race/ethnicity (*P* = .01), BMI category (*P* < .001), education level (*P* < .001), neighborhood income level (*P* = .006). Smoking status (*P* = .07) was only marginally associated with diabetes incidence, and sex (*P* = .36) was not associated with diabetes incidence. Age, race/ethnicity, BMI category, education level, neighborhood income, and smoking were considered for the final multivariable model. All with the exception of neighborhood income level and smoking status were found to be independent predictors of incident diabetes. Diabetes incidence was significantly higher among participants aged 45 or older than among those aged 18 to 44, among non-Hispanic blacks and Hispanics than among whites, among overweight and obese people than among normal-weight people, and among people with less than a high school diploma than among those with more than a high school diploma (Table 2).

**Table 2:** Factors Associated With the Overall Estimated Incidence of Self-Reported Diabetes, New York City Community Health Survey, Combined 2002, 2004, and 2008^a^

Characteristic	Multivariable-Adjusted OR (95% CI)
**Age, y**	
18–44	1 [Reference]
45–64	4.26 (2.87–6.33)
≥65	4.26 (2.76–6.59)
**Race/ethnicity**	
Non-Hispanic white	1 [Reference]
Non-Hispanic black	1.65 (1.10–2.46)
Hispanic	1.78 (1.13–2.80)
Other	1.94 (1.17–3.20)
**BMI category^b^**	
Normal	1 [Reference]
Overweight	1.62 (1.06–2.47)
Obese	3.02 (2.02–4.53)
**Education**	
>High school graduate (reference)	1 [Reference]
High school graduate	1.36 (0.94–1.97)
<High school graduate	1.71 (1.09–2.68)

Abbreviations: OR, odds ratio; CI, confidence interval; BMI, body mass index.

^a^ Multivariable analysis included only those variables with a *P* < .10 in the bivariate analysis (age, race/ethnicity, BMI, education level, neighborhood income, and smoking). In the multivariable analysis, neighborhood income and smoking were not significant and, therefore, were excluded from the final model.

^b^ BMI was calculated as weight in kilograms divided by height in meters squared, and participants were classified into BMI categories (under/normal weight = BMI <25.0; overweight = BMI 25.0–29.9; obese = BMI ≥30.0) (13).

## Discussion

This study suggests that diabetes incidence is stabilizing in New York City. After adjusting for multiple factors, older age and obesity had the strongest association with diabetes incidence. Furthermore, being non-Hispanic black or Hispanic and having a lower education level were identified as significant predictors of diabetes incidence in the study period. Our findings will be useful not only to NYC diabetes prevention efforts but also to inform decision making in other urban, ethnically diverse areas in the United States.

These findings can be compared with other cross-sectional studies that used similar methods with survey data to calculate incidence and to identify newly diagnosed cases of disease. A study using BRFSS, which is the most similar to CHS in terms of survey methods, documented an overall estimated age-adjusted national incidence of 9.1 per 1,000 in 2005 through 2007 (5). Similarly, data from the National Health Interview Survey (NHIS) show an age-adjusted national diabetes incidence of 8.5 per 1,000 population in 2008 (20). Our estimates of 2008 NYC incidence were similar to both 2005–2007 BRFSS data and 2008 NHIS data. Furthermore, risk factors that we found to be independently associated with diabetes incidence were similar to those previously reported by Geiss et al using NHIS data, which are collected using an in-person interview (6).

Although the incidence for 2008 was similar between local and national estimates, the direction of the observed trends over the years was different. BRFSS data show that the average national age-adjusted incidence of diabetes increased 90% from 4.8 per 1,000 population in 1995–1997 to 9.1 per 1,000 population in 2005–2007 (5). Similarly, national data from NHIS show that age-adjusted diabetes incidence increased from 7.1 to 8.5 per 1,000 population from 2002 to 2008 (20).

Our study found that the age-adjusted incidence estimates increased in NYC from 9.4 to 11.9 per 1,000 population from 2002 to 2004 and then decreased subsequently to 8.6 per 1,000 in 2008 rather than continuing to increase, as seen nationally. This coincides with a local trend of decreasing childhood obesity (21) and a stabilization of obesity among adults (11). It may be that NYC is seeing a shift in these chronic diseases, ahead of the rest of the country. Although this trend may be heralding a true stabilization in diabetes incidence in NYC, 2 alternate scenarios related to the survey methodology should be considered. The first is the smaller sample size in 2008 compared with 2002 and 2004, which may have prevented us from detecting significant increases in incidence. The second is the change in survey questions between the first 2 years of study and the third (16–18), which could be masking a true increase in incidence if people answered the questions differently. Although the questions did not change drastically, we cannot rule out this possibility.

The key strength of this study is that it provides a population-based incidence estimate for a large jurisdiction. However, several limitations should be noted. First, the cross-sectional data limited our ability to assess the temporal relationship between risk factors and diabetes incidence. Second, because the diagnosis of diabetes was self-reported by respondents, survey responses regarding the diabetes diagnosis and the date of diagnosis were subject to recall bias and misinterpretation. However, this bias is unlikely to have affected the trend analysis reported here, as we have no reason to think it has changed over time. Third, CHS diabetes incidence data were collected only for 3 years, which may have restricted the ability to detect trends. Additional years of data are needed to detect a significant change in the overall incidence. Fourth, institutionalized adults and those without residential telephones were not represented in the sampling frame, limiting the generalizability of our findings. However, in 2008 a pilot study of CHS, which included cell phone users, showed that diabetes prevalence was similar between landline and combined landline and cell phone–only samples (22). Fifth, since the CHS was conducted during different times of the year for the 3 years of the study (2002: May to July 2002; 2004: May 2004 to February 2005; 2008: September 2008 to February 2009), survey responses may be influenced by seasonal variation. Furthermore, although physical inactivity is a risk factor for diabetes, we did not have a variable that allowed us to examine this factor temporally because the CHS question pertaining to physical activity assesses activity during the previous 30 days only.

The number of new diabetes cases increased significantly over the past 2 decades in the United States, and continued efforts at the national, state, and local levels to alter this trend are necessary. Our findings suggest that this trend in NYC may be slowing. To maintain this trend and achieve further progress, we must ensure that effective prevention programs and policies, focusing on weight as the most modifiable risk factor for developing diabetes, are in place. Lifestyle modification programs, which can decrease diabetes incidence by up to 58% and can be cost-effective, should be widely implemented (23–25), as should policies and practices focusing on sustainable environmental changes to facilitate healthy eating and physical activity. Large-scale prevention efforts should be implemented to maintain this trend in stabilization and aim for reversing the diabetes epidemic in the coming years.
